# Supplementary material for: The Molecular Chaperone TCP1 Affects Carcinogenicity and Is a Potential Therapeutic Target for Acute Myeloid Leukemia
Source: Pharmaceutics. 2025 Apr 24;17(5):557. doi: 10.3390/pharmaceutics17050557 (PMC12114683; doi:10.3390/pharmaceutics17050557)
Supplement: Supplementary file 1 [file pharmaceutics-17-00557-s001.zip › pharmaceutics-3501359-supplementary.pdf]

Supplementary Figures

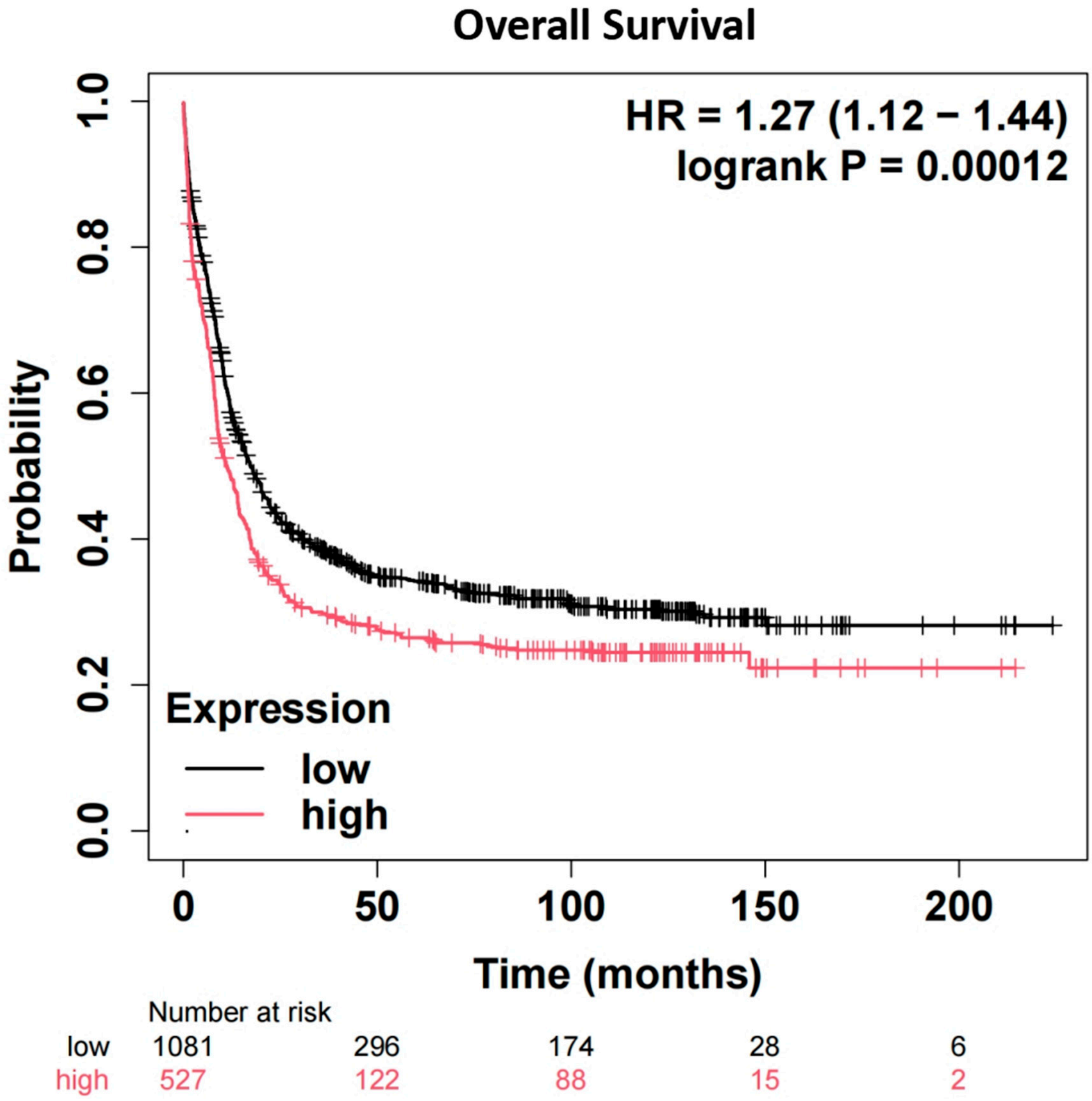

Figure S1. Kaplan-Meier overall survival curves for AML patients stratified by TCP1 expression.

A

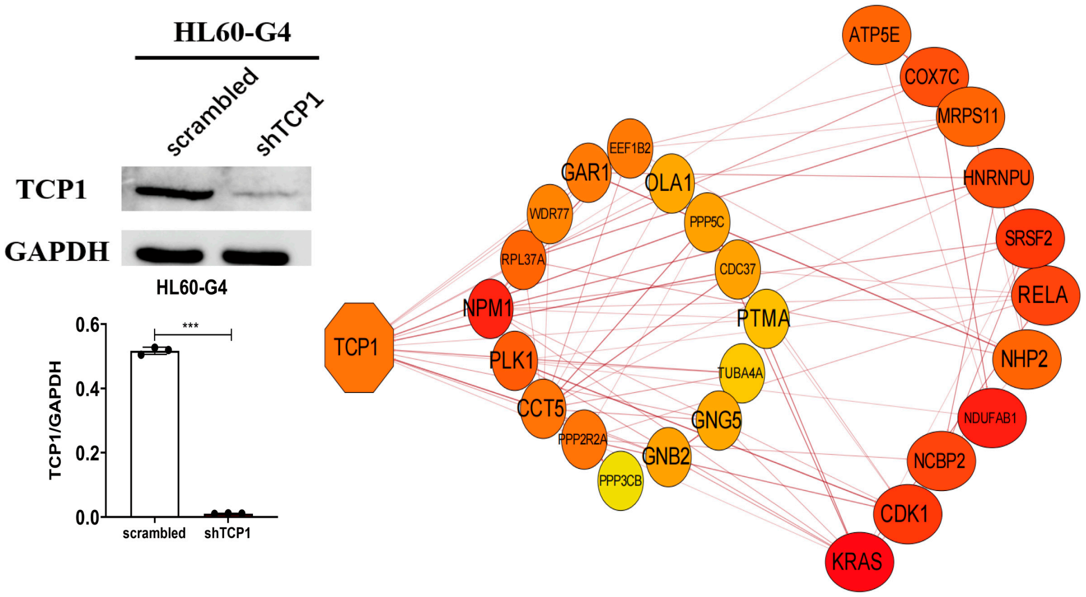
HL60-G4
scrambled
shTCP1
TCP1
GAPDH
HL60-G4
***
TCP1/GAPDH
0.6
0.4
0.2
0.0
scrambled
shTCP1
TCP1
ATP5E
COX7C
MRPS11
HNRNPU
SRSF2
RELA
NHP2
NDUFAB1
NCBP2
CDK1
KRAS
EEF1B2
GAR1
OLA1
WDR77
PPP5C
RPL37A
CDC37
NPM1
PTMA
PLK1
TUBA4A
CCT5
GNG5
PPP2R2A
GNB2
PPP3CB

B

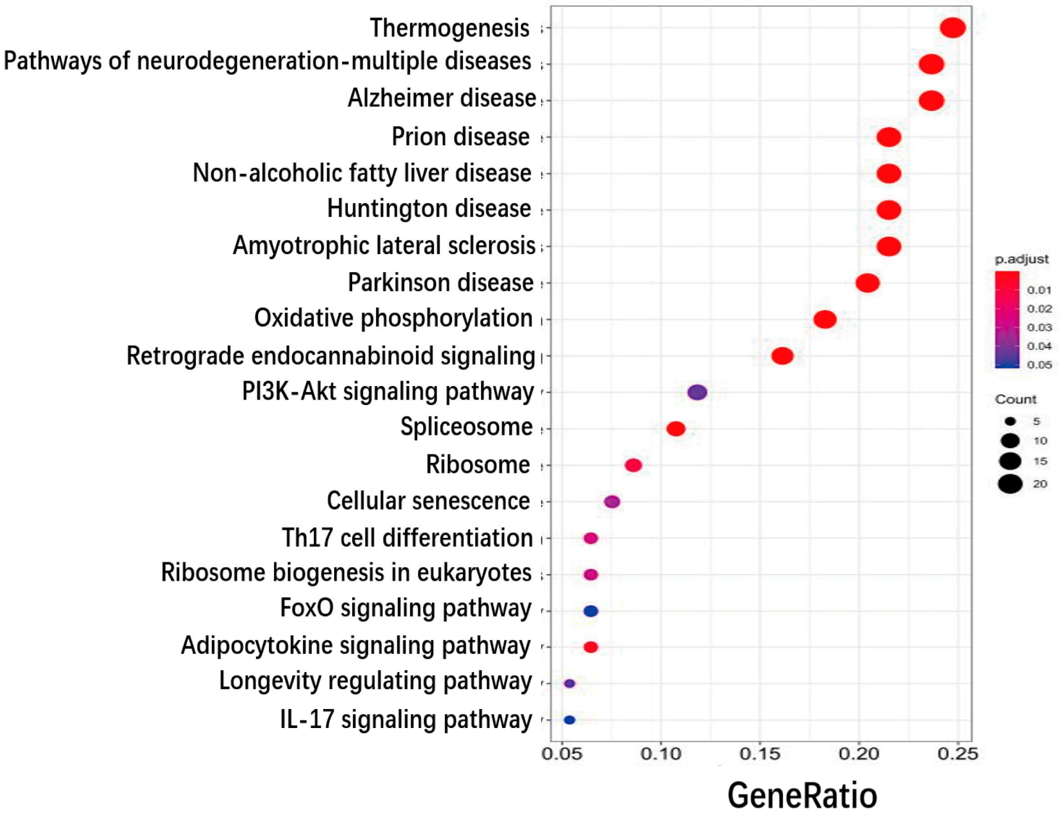
Thermogenesis
Pathways of neurodegeneration-multiple diseases
Alzheimer disease
Prion disease
Non-alcoholic fatty liver disease
Huntington disease
Amyotrophic lateral sclerosis
Parkinson disease
Oxidative phosphorylation
Retrograde endocannabinoid signaling
PI3K-Akt signaling pathway
Spliceosome
Ribosome
Cellular senescence
Th17 cell differentiation
Ribosome biogenesis in eukaryotes
FoxO signaling pathway
Adipocytokine signaling pathway
Longevity regulating pathway
IL-17 signaling pathway
0.05
0.10
0.15
0.20
0.25
GeneRatio
p.adjust
0.01
0.02
0.03
0.04
0.05
Count
5
10
15
20

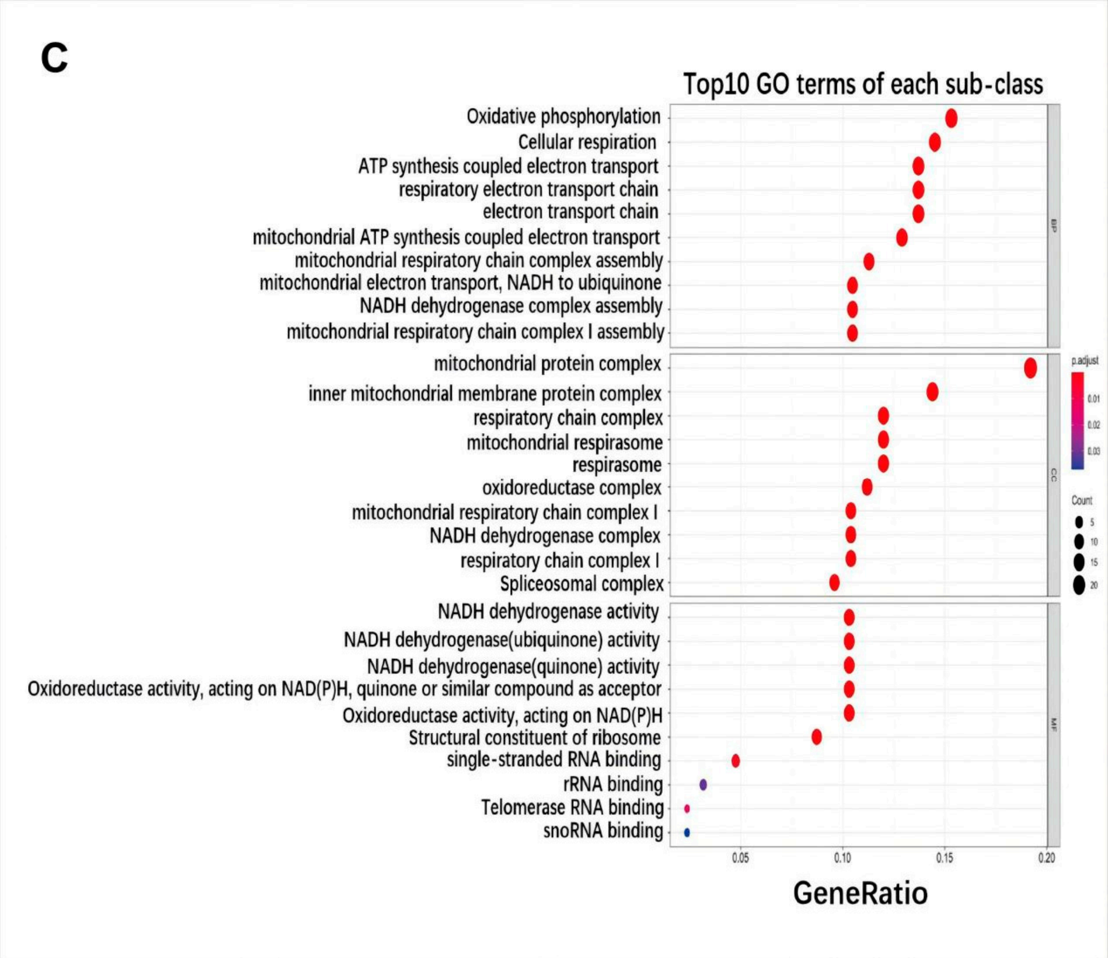

Figure S2. TCP1 may regulate multiple signaling pathways related to AML cell survival. (A) Validation of TCP1 expression in the HL60-G4 cell line after TCP1 knockdown by Western blot; The PPI network of TCP1 in AML was obtained from the iTRAQ relative quantitative proteomics and was arranged according to degree value. **(B)** Scatter plot of KEGG enrichment of overlapping targets. **(C)** Scatter plot of GO function enrichment of overlapping targets. The X-axis indicates the GeneRatio enriched in this pathway. The redder the color, the smaller the p. adjust value, it also indicates the reliability and importance. The bluer the color, the greater the p. adjust value.

Supplementary Tables

Table S1. The IC_{50} values of compounds in AML cell lines

Compound	IC_{50} (μM) in cell growth inhibition	
	HL-60	HL-60-G4
FTY720	4.35	3.20
NSC348884	1.33	0.73
Cediranib	8.84	5.59
Foretinib	3.50	2.70
Lipitor	9.40	8.90
